# Supplementary material for: Diurnal control of iron responsive element containing mRNAs through iron regulatory proteins IRP1 and IRP2 is mediated by feeding rhythms
Source: Genome Biol. 2024 May 21;25:128. doi: 10.1186/s13059-024-03270-2 (PMC11106963; doi:10.1186/s13059-024-03270-2)
Supplement: Supplementary file 1 — Additional file 1: Supplementary Figures. This file (.pdf) contains Figures (and legends) S1 and S2. Fig. S1 shows IRE hairpin sequences and predicted secondary structures of rhythmically and non-rhythmically regulated mRNAs (related to Fig. 1). Fig. S2 shows liver single-cell sequencing data that validates hepatocyte expression of Alas2 (related to Fig. 1). [file 13059_2024_3270_MOESM1_ESM.pdf]

Additional file 1: Figures S1-S2

Diurnal control of iron responsive element containing mRNAs through iron regulatory proteins IRP1 and IRP2 is mediated by feeding rhythms

Hima Priyanka Nadimpalli[1,#], Georgia Katsioudi[1,#], Enes Salih Arpa[1,#], Lies Chikhaoui[1], Alaaddin Bulak Arpat[1], Angelica Liechti[1], Gaël Palais[2], Claudia Tessmer[3], Ilse Hofmann[3], Bruno Galy[2], David Gatfield[1]*

Affiliations:

[1] Center for Integrative Genomics, University of Lausanne, 1015 Lausanne, Switzerland.

[2] German Cancer Research Center (DKFZ), Division of Virus-associated Carcinogenesis, Im Neuenheimer Feld 280, 69120, Heidelberg, Germany.

[3] German Cancer Research Center (DKFZ), Core Facility Antibodies, Im Neuenheimer Feld 280, 69120, Heidelberg, Germany.

email addresses: himapriyanka.nadimpalli@unil.ch, georgia.katsioudi@unige.ch, enessalih.arpa@unil.ch, lies.chikhaoui@outlook.fr, bulak.arpat@gmail.com, angeliechti@hotmail.com, g.palais@dkfz-heidelberg.de, c.tessmer@dkfz-heidelberg.de, i.hofmann@dkfz.de, b.galy@dkfz-heidelberg.de, david.gatfield@unil.ch

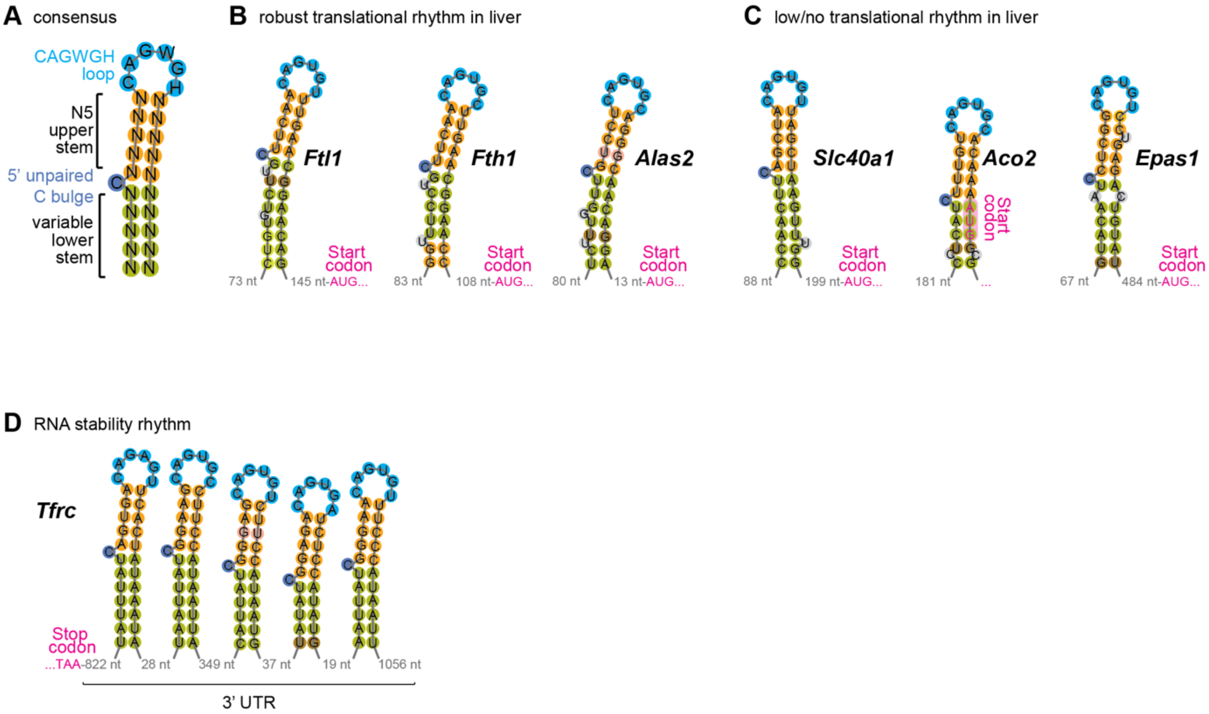

Figure S1. IRE hairpin sequences and predicted secondary structures of rhythmic and non-rhythmic mRNAs.

(**A**) Schematic of the IRE hairpin consensus sequence and structure. IREs are about 35 nt in length land are characterized by an apical 6-nt loop motif 5'-CAGWGH-3' (W = A or T and H = A, C or T), and two stem regions (N5 upper stem and variable lower stem) that are separated by a unpaired C bulge. Colour code depicts sequence conservation across IREs from high (blue) to medium (orange) and low (olive).

(**B**) Sequence and predicted structure, as well as relative position within 5' UTR, of the IREs of the murine transcripts *Ftl1, Fth1* and *Alas2* for which the gene expression profiles are shown in main Fig. 1C. Color code as in (A).

(**C**) Sequence and predicted structure, as well as relative position within 5' UTR, of the IREs of the murine transcripts *Slc40a1, Aco2* and *Epas1* that are shown in main Fig. 1D. Color code as in (A).

(**D**) Sequence and predicted hairpin structures of the five IREs annotated within the mouse *Tfrc* 3' UTR. Nucleotide distances from the TAA stop codon, between individual haipins, and to the annotated transcript end are noted in grey. Color code as in (A).

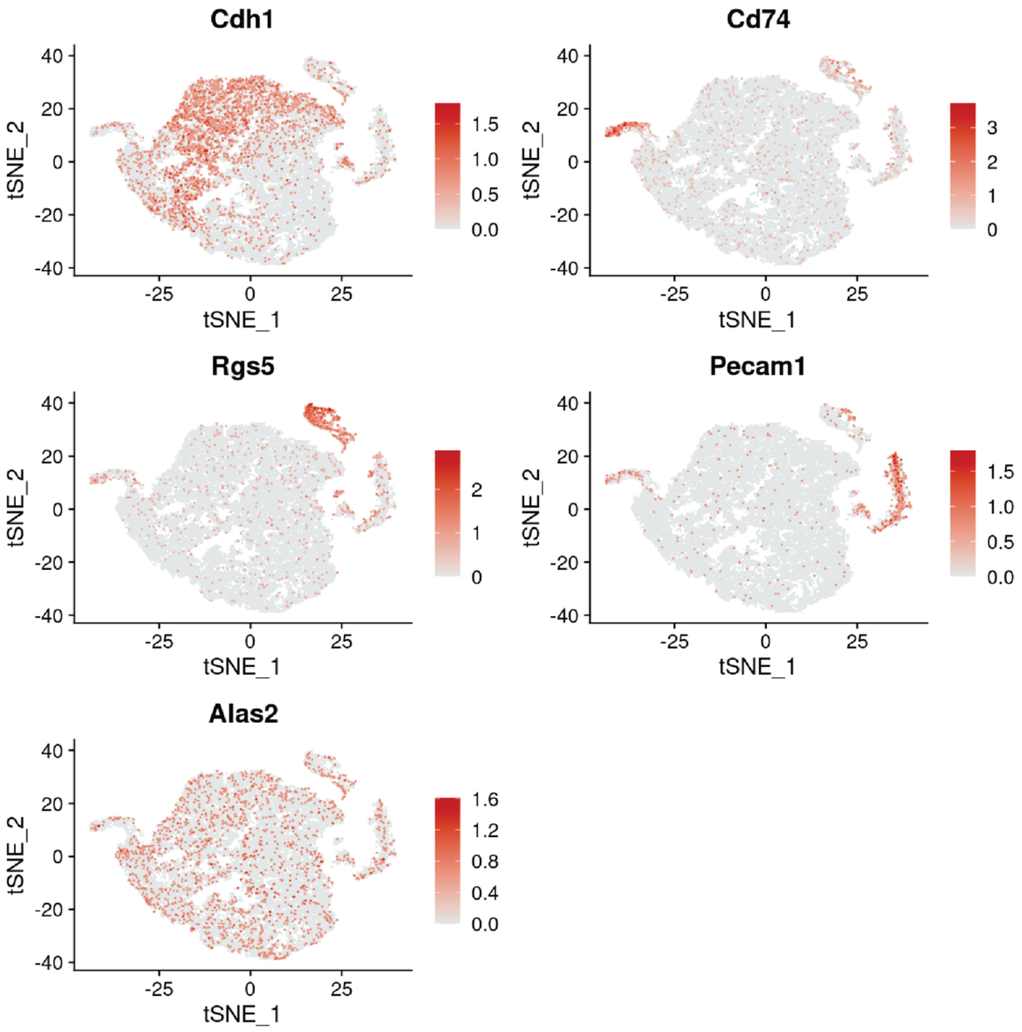

Figure S2. Single-cell sequencing data validates hepatocyte expression of *Alas2*.
t-SNE plot of liver single cell RNA-seq analysis using data from Martini et al., bioRxiv 2023.10.07.561324. The large central group of cells are hepatocytes. As marker transcripts in the first 4 plots, *Cdh1* is specific for periportal hepatocytes; *Cd74* shows immune cells; *Rgs5* identifies stellate cells; *Pecam1* are endothelial cells. *Alas2* is lowly expressed everywhere, including across periportal (*Cdh1*-positive) and pericentral (*Cdh1*-negative) hepatocytes. Figure preparation acknowledgement: Tomasz Martini, EPF Lausanne, Switzerland.
