# Supplementary material for: Diurnal control of iron responsive element containing mRNAs through iron regulatory proteins IRP1 and IRP2 is mediated by feeding rhythms
Source: Genome Biol. 2024 May 21;25:128. doi: 10.1186/s13059-024-03270-2 (PMC11106963; doi:10.1186/s13059-024-03270-2)

Additional file 3: Uncropped immunblot scans

Related to publication:

Diurnal control of iron responsive element containing mRNAs through iron regulatory proteins IRP1 and IRP2 is mediated by feeding rhythms

Hima Priyanka Nadimpalli[1,#], Georgia Katsioudi[1,#], Enes Salih Arpa[1,#], Lies Chikhaoui[1], Alaaddin Bulak Arpat[1], Angelica Liechti[1], Gaël Palais[2], Claudia Tessmer[3], Ilse Hofmann[3], Bruno Galy[2], David Gatfield[1]*

Affiliations:

[1] Center for Integrative Genomics, University of Lausanne, 1015 Lausanne, Switzerland.

[2] German Cancer Research Center (DKFZ), Division of Virus-associated Carcinogenesis, Im Neuenheimer Feld 280, 69120, Heidelberg, Germany.

[3] German Cancer Research Center (DKFZ), Core Facility Antibodies, Im Neuenheimer Feld 280, 69120, Heidelberg, Germany.

email addresses: himapriyanka.nadimpalli@unil.ch, georgia.katsioudi@unige.ch, enessalih.arpa@unil.ch, lies.chikhaoui@outlook.fr, bulak.arpat@gmail.com, angeliechti@hotmail.com, g.palais@dkfz-heidelberg.de, c.tessmer@dkfz-heidelberg.de, i.hofmann@dkfz.de, b.galy@dkfz-heidelberg.de, david.gatfield@unil.ch

Figure 2E

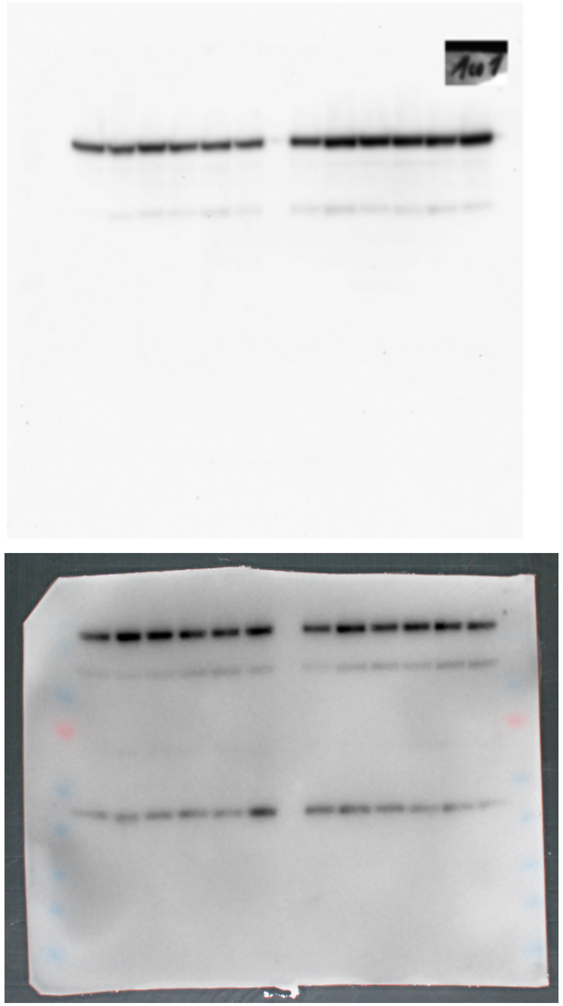

ACO1

VINCULIN

140 kDa
115 kDa

Figure 2G

IREB2

VINCULIN

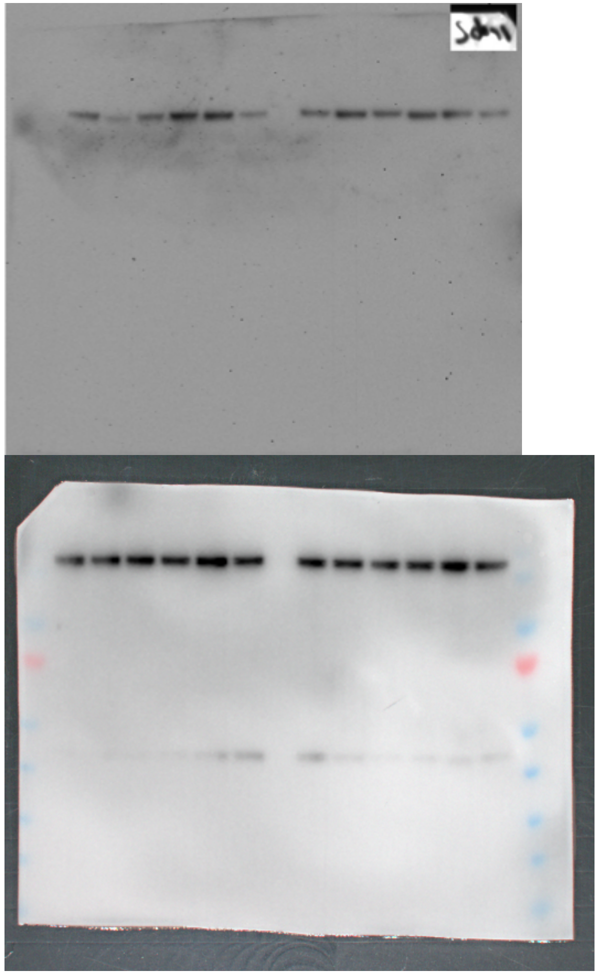

140 kDa

115 kDa

Figure 2K

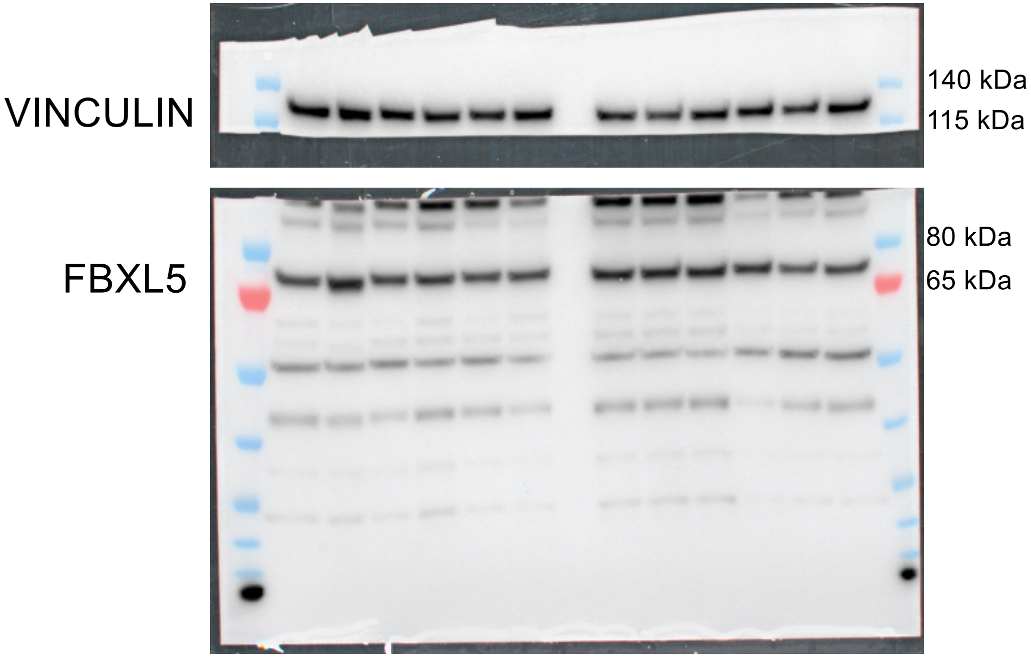

Figure 2I

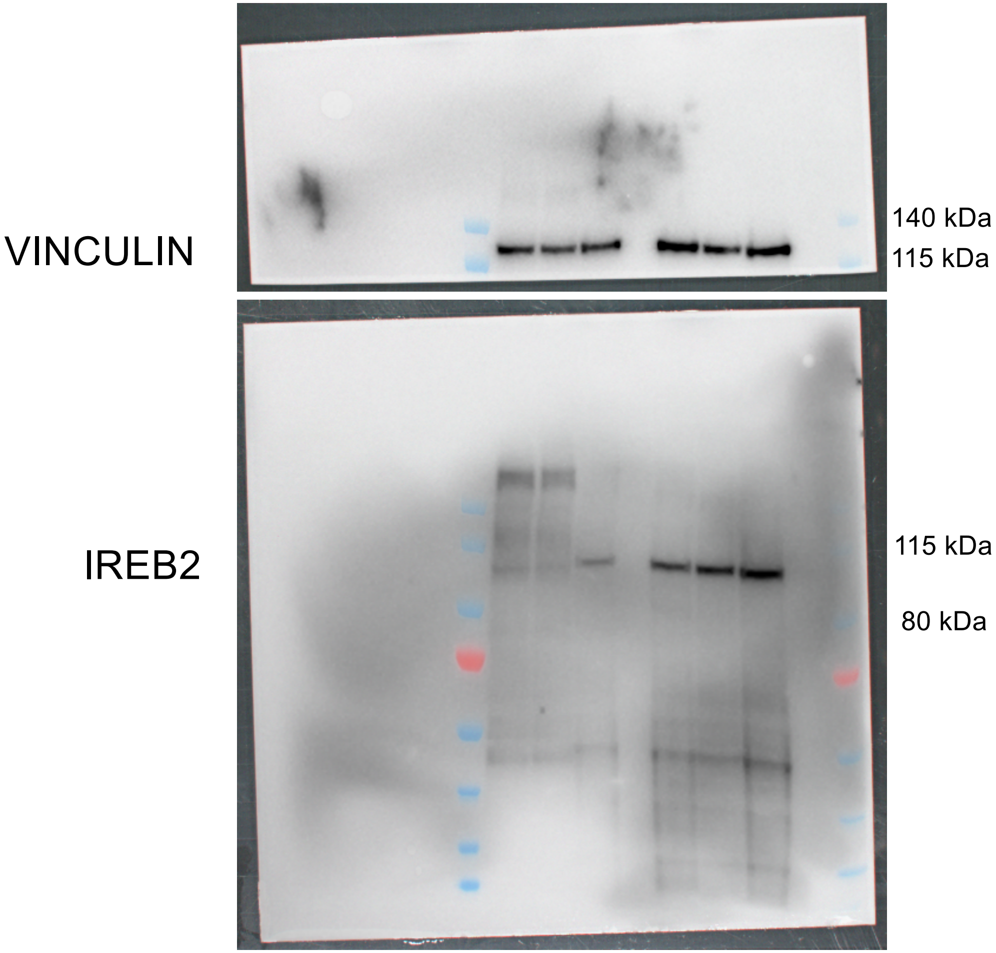

Figure 4G

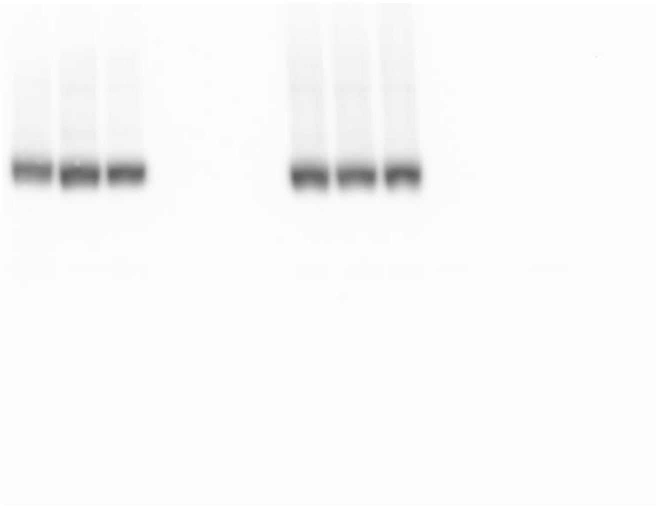

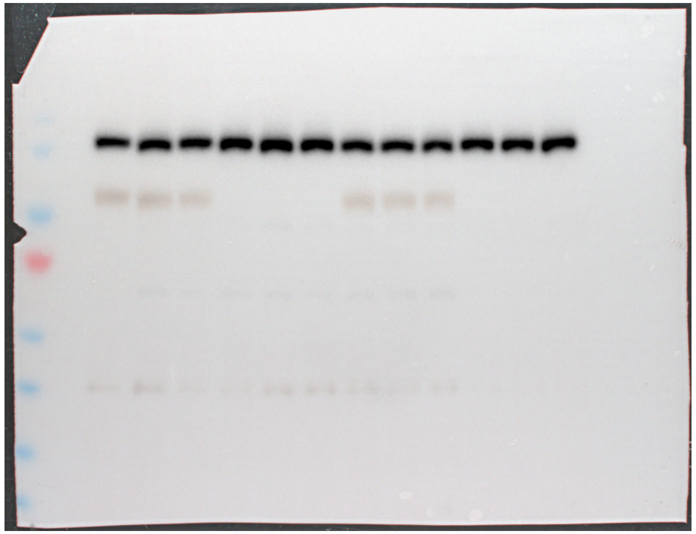

Figure 4G

IREB2/IRP2

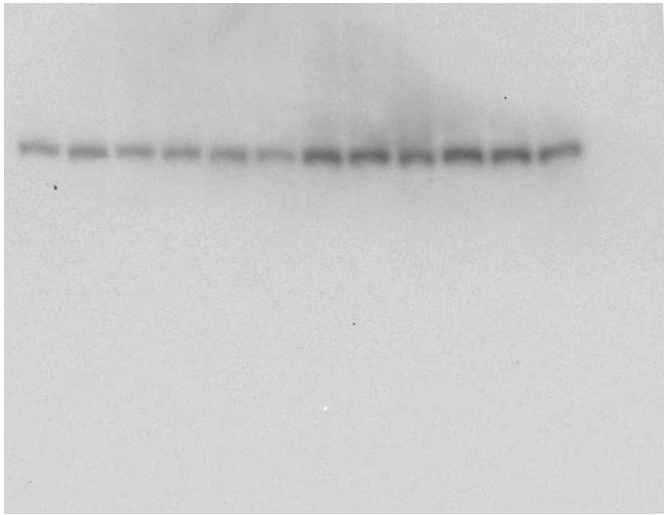

140 kDa
115 kDa

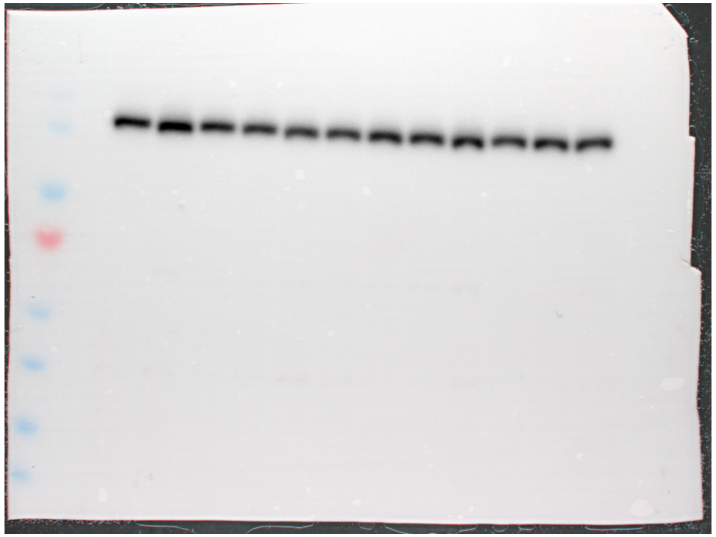

VINCULIN

Figure 4J

ACO1/IRP1

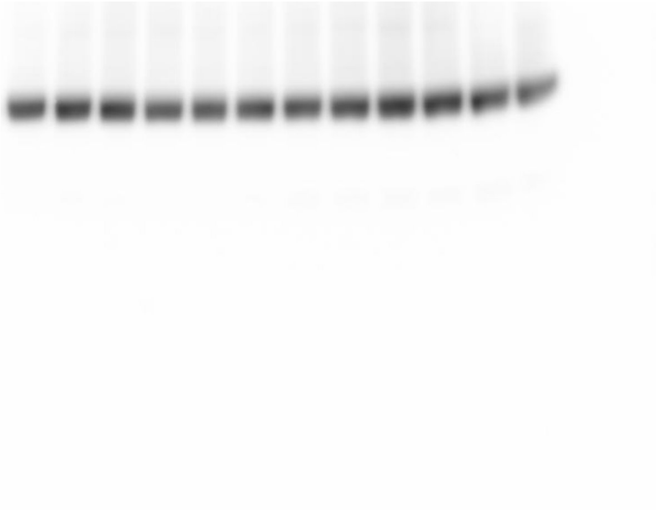

140 kDa
115 kDa

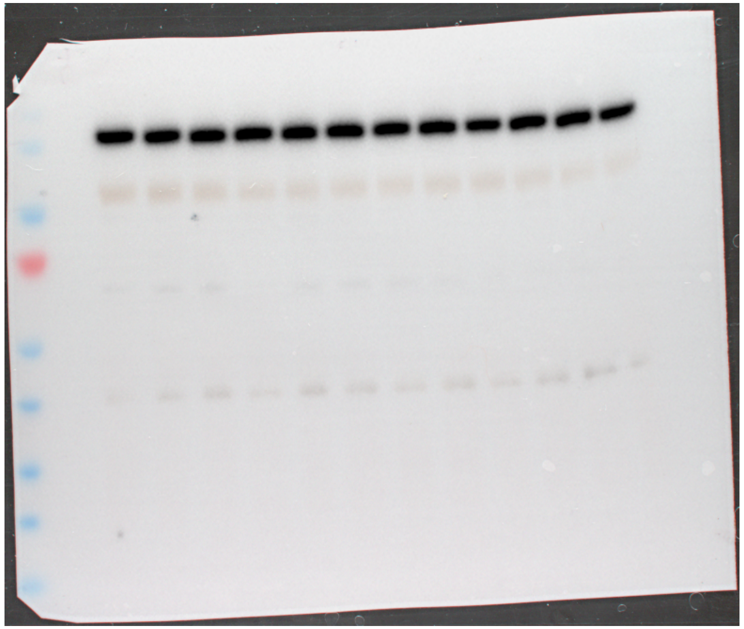

VINCULIN

Figure 4J

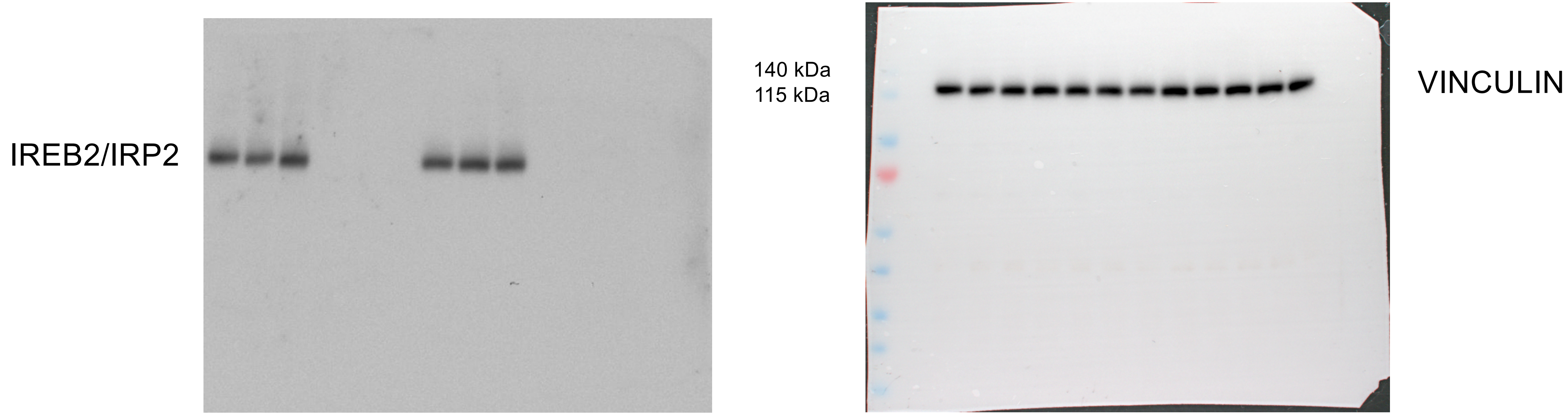

Supplement: Supplementary file 3 — Additional file 3. Uncropped immunoblots. This file (.pdf) contains the uncropped immunoblot images from this study. Related to Fig. 2 and Fig. 4. [file 13059_2024_3270_MOESM3_ESM.pdf]
