# Supplementary material for: Diurnal control of iron responsive element containing mRNAs through iron regulatory proteins IRP1 and IRP2 is mediated by feeding rhythms
Source: Genome Biol. 2024 May 21;25:128. doi: 10.1186/s13059-024-03270-2 (PMC11106963; doi:10.1186/s13059-024-03270-2)
Supplement: Supplementary file 4 — Additional file 4. Peer review history. [file 13059_2024_3270_MOESM4_ESM.docx]

**Review history**

**First round of review**

**Reviewer 1**

**Comments to author:**

Review Commons *Report 1*.

**Summary**

This paper provides evidence for the diurnal regulation of specific subset of iron regulatory elements (IREs)-containing mRNAs in liver by iron regulatory proteins 1 and 2 (IRP1 and IRP2) in mice. The authors show that IRP2 oscillates over 24 h period to regulate IRE-containing mRNAs in the light phase, and collaborates with IRP1 to regulate IRE-mRNAs in the dark phase.

**Major Comments**

The authors have carefully performed experiments, and convincingly show that 5'-IRE containing transcripts (Fth1, Ftl1, Fpn and Alas2) display significant amplitude rhythms in ribosome occupancy in liver. Tfrc mRNA, which harbors a 3' IRE, also showed a rhythmic pattern in both liver and kidney. The changes in IRE-containing mRNAs correlated with IRP2 protein abundance. Further studies performed using Aco1 and Ireb2 knockout mice showed that both IRP1 and IRP2 are required for rhythmic regulation of IRE-containing mRNAs. Overall, the findings in this paper are interesting and novel, and show for the first time that IRE-containing mRNAs required for maintenance of cellular iron metabolism and IRP2 are subjected to rhythmic regulation.
Alas2 is expressed mainly in erythroid cells and not liver, whereas Alas1 is ubiquitously expressed. Therefore, it is possible that Alas2 in this study may originate from red cells/reticulocytes in the
liver, and not from hepatocytes.

**Minor Comments**

The paper is dense and not easy to read. For example, the section on Tfrc regulation and NMD regulation is lengthy and perhaps not necessary for the paper and the section on "Previous observations in IRE-IRP regulation...." could be included in the discussion rather in than in the Results section. Some figures could be included in a supplement. A description of the new anti-IREB2 antibody is needed. What IRP2 sequence was used to generate antibodies? A model summarizing the data would be useful.

**Referees cross-commenting**

I agree with Reviewer 2 that the first sections in the manuscript are lengthy and not needed. More importantly, clues to the mechanism (e.g. iron, heme) regulating the rhythmic translation of IRP1 and IRP2 IRE-mRNAs in liver would increase the significance of the work. Overall, the findings are novel, and would be of interest to the iron metabolism and circadian rhythm fields.

**Reviewer 2**

**Comments to author:**

Review Commons *Report 2*

In the manuscript entitled "Diurnal control of iron responsive element (IRE)-containing mRNAS through iron regular proteins IRP1 and IRP2 is mediated by feeding rhythms", Nadimpalli et al. uncover and mechanistically dissect how the circadian clock and feeding regulates the expression of proteins involved in iron homeostasis in mice. The authors first utilized RNAseq and ribose data and found that a subset of mRNAs containing IREs display rhythmic translation in the liver and/or kidney. The authors then utilized previously published or newly generated datasets to study the origin of these oscillations. After a careful and thoughtful examination, they determine that the oscillations of those mRNAs in the liver are mainly driven by feeding-associated signals, although they are influenced by other factors.
This is a very careful and detailed study that addresses an important issue. The study is really rigorous and the logic is very well explained. So overall this study is very solid and the main conclusion of the study (that the oscillations of those mRNAs are driven by feeding) is solidly established. However, the study falls short on addressing the mechanism of this regulation and if it is different of other feeding regulated mRNA oscillations. This diminishes the significance of the study unless additional mechanistic details are provided. Also, the manuscript first sections (which mainly describe negative results) seem too long and descriptive. Still this is an important and solid study.

**Reviewer 3**

**Comments to author:**

Review Commons *Report 3*

The present manuscript highlights the previously neglected component of diurnal rhythms into the study of iron regulation in the liver, a key organ in the systemic regulation of the metal. A major, well substantiated finding is that IRP1 takes over from IRP2 as a highly relevant regulator during a time of maximal use of the combined system. The presence of a "dietary signal" that sustains the cycling of cellular IRE binding activity in the liver, although undisputable, is perhaps a lesser claim until these or other investigators can confirm or refute the possibility that iron itself (i.e., the best-established factor affecting cellular IRE-binding) is such a signal. If iron does the job, then the interest lies in showing diurnal rhythmicity of its availability in the circulatory system, presumably linked to dietary iron absorption. There is plenty of clinical evidence of this in humans, but the present study along with the cited preprint by Bennett et al. appear to be the first demonstrations of this diurnal variation in mice.

The manuscript has other strengths not immediately evident from the above claims made in the abstract. It contains an elegant balance of reanalyzing and reassessing "big data" produced by the same laboratory in the past in light of new experimental findings that have appeared in the meantime in the literature together with important new additions of such data from combined RNAseq and ribo-seq collections using IRP1 and IRP2 knockout animals and, importantly, by making use of published material from other studies that have provided relevant comparators from other knockouts that studied aspects of liver circadian biology. The approach, besides providing robust testing of the ideas presented, opens the field to questions that remain unanswered but seem highly relevant (I come to these below). The authors write in a very open manner not only about the new findings but also about aspects we do not understand, and their systems biology approach is likely to generate new hypotheses to address incognita.

Let me therefore be clear upfront that the response that follows is written on the premise that I evaluate the work presented as ready for publication: The figures have been constructed with care summarizing a lot of careful investigations and the main conclusions derive seamlessly from the experimental data. Rather than taken as potential criticism to the authors, I would ask that the counterviews or limitations that may arise from my response to the paper are better taken as a celebration of the work - and views - presented. Such a discussion is only possible due to the open style of this communication mentioned above and is meant to provoke a dialogue or even drive further questioning of the datasets and the design of future experimental approaches. If the authors find any of these comments useful for their revision, they are welcome to take them onboard, but everything that follows should be read under the term "optional".

One nuance in the field of circadian biology is that a rhythm is deemed to be genuinely "circadian" when it continues in the absence of zeitgebers. In this sense, although all experiments are valuable, the "collapse" of the rhythm in the paradigms where dietary rhythms have been disrupted makes the phenomenology a candidate "epiphenomenon" rather than being closer related to the biological clock(s). Likewise, in the manuscript we never learn how the liver IRE-binding activity behaves in constant darkness. Where the authors mention in a parenthesis "moreover, there are documented links between iron and the circadian timekeeping mechanism itself", I invite them to take a closer look to the paper Konstantinos Mandilaras and I coauthored in 2012 "Genes for iron metabolism influence circadian rhythms in Drosophila melanogaster". In that work, we showed that RNA interference of genes that are required for iron sulfur cluster formation (including on IRP1) in the central clock neurons of the fly result in loss of the circadian rhythm when flies were kept at constant darkness (not so when they were kept under light:dark oscillation). So this point should probably remain open.

Given the phenotypes collected from RNAi in different cell types, we became sensitive to the notion that different cell types may work with different sets of what we might call collectively "iron metabolism genes". Thus, while reading the present differences between the relative contributions of IRP1 and IRP2 in mouse liver at different times during the day (and night), I kept wondering if both function in the hepatocytes or whether the macrophages or other cell types in the liver may have their own particular contributions.

Another issue raised by the authors early on relates to the differential effects of IRP1/2 "activation" on different IRE-containing transcripts. This is a fascinating problem, not answered by the six transcripts shown in figure 1G, but I consider that the present paper offers a great service to the field in figure 5I, where an even more comprehensive grouping of IRE-containing transcripts is provided in terms of their "regulation" by IRPs. Future research should attempt to discover features that correlate within the sets of transcripts, as grouped in here.

Thus, another point made repeatedly by the authors that despite four decades of work on the IRPs we still have open questions about how they "regulate" is well taken. In the same tone, their results in relation to IRP2 degradation show that the story of how the presence of iron leads to the degradation of IRP2 has not been fully elucidated, either.

**Referees cross-commenting**

Reading the comment from Reviewer #2 over the lack of a mechanism to explain why only four transcripts with IREs amongst a larger pool are subject to circadian regulation by IRPs somehow reduces the significance of the study, one has to agree that a discovery - likely another component in the system - is wanting. I remain of the view that the present work exposes this "weakness" of the entire field in a global as opposed to a partial manner and in doing so, makes a significant contribution, especially by further sub-classifying the IRE-containing transcripts according to their responsiveness in the diurnal occupancy of their IREs.

I would like to reinforce the comment of reviewer 1 with respect to the antibodies used in this study that should be made available to the community, given the specificity described. My congratulations to the authors.

**Authors’ response to reviewers**

# Reviewer #1 (Evidence, reproducibility and clarity (Required)):

Summary

This paper provides evidence for the diurnal regulation of specific subset of iron regulatory elements (IREs)-containing mRNAs in liver by iron regulatory proteins 1 and 2 (IRP1 and IRP2) in mice. The authors show that IRP2 oscillates over 24 h period to regulate IRE-containing mRNAs in the light phase, and collaborates with IRP1 to regulate IRE-mRNAs in the dark phase.

Major Comments

The authors have carefully performed experiments, and convincingly show that 5'-IRE containing transcripts (Fth1, Ftl1, Fpn and Alas2) display significant amplitude rhythms in ribosome occupancy in liver. Tfrc mRNA, which harbors a 3' IRE, also showed a rhythmic pattern in both liver and kidney. The changes in IRE-containing mRNAs correlated with IRP2 protein abundance. Further studies performed using Aco1 and Ireb2 knockout mice showed that both IRP1 and IRP2 are required for rhythmic regulation of IRE-containing mRNAs. Overall, the findings in this paper are interesting and novel, and show for the first time that IRE-containing mRNAs required for maintenance of cellular iron metabolism and IRP2 are subjected to rhythmic regulation.

**Author response:** We thank the Reviewer for the positive evaluation of our manuscript.

Alas2 is expressed mainly in erythroid cells and not liver, whereas Alas1 is ubiquitously expressed. Therefore, it is possible that Alas2 in this study may originate from red cells/reticulocytes in the liver, and not from hepatocytes.

**Author response:** We would like to thank the Reviewer for the comment that is indeed pertinent. It is well established that *Alas1* is the main transcript encoding delta-aminolevulinate synthase activity in hepatocytes, and *Alas2* is about 10-fold less abundant in total liver RNA-seq data (quantified from own RNA-seq data, not shown).

We are nevertheless relatively sure that the Alas2 signal comes from low expression in hepatocytes; the best argument in support of this is the analysis of single-cell RNA-seq data, as shown in the following Rebuttal Figure 1, which we have also included in the revised version of the manuscript as Supplementary Figure S2.

**Rebuttal Figure 1:** Liver single cell RNA- seq analysis using data from Martini et al., bioRxiv 2023.10.07.561324.

In the t-SNE plot, the large central group of cells are hepatocytes. As marker transcripts in the first 4 plots, *Cdh1* is specific for periportal hepatocytes; *Cd74* shows immune cells; *Rgs5* identifies stellate cells; *Pecam1* are endothelial cells. *Alas2* is lowly expressed everywhere, including across periportal (*Cdh1*-positive) and pericentral (*Cdh1*- negative) hepatocytes.

Figure preparation acknowledgement: Tomasz Martini, EPF Lausanne, Switzerland.

Minor Comments

The paper is dense and not easy to read. For example, the section on Tfrc regulation and NMD regulation is lengthy and perhaps not necessary for the paper and the section on "Previous observations in IRE-IRP regulation	" could be included in the discussion rather in than in the Results

section. Some figures could be included in a supplement. A description of the new anti-IREB2 antibody is needed. What IRP2 sequence was used to generate antibodies? A model summarizing the data would be useful.

**Author response:** In the revised version, we have worked on simplifying the manuscript. As by the Reviewer’s suggestion, we have removed the section of *Tfrc* and NMD completely, and we have also restructured other parts, including the section “Previous observations in IRE-IRP regulation…” that has been removed from the Results part and integrated in a shortened form into the Discussion. Several figure panels, in particular from Figure 1 and 2, have been moved to supplement. A description of the antibodies and the antigen used for immunization can now be found in the Materials and Methods section. Finally, in Figure 6F, we provide a model that summarizes our findings.

**Referees cross-commenting**

I agree with Reviewer 2 that the first sections in the manuscript are lengthy and not needed. More importantly, clues to the mechanism (e.g. iron, heme) regulating the rhythmic translation of IRP1 and IRP2 IRE-mRNAs in liver would increase the significance of the work. Overall, the findings are novel, and would be of interest to the iron metabolism and circadian rhythm fields.

**Author response:** The first sections of the manuscript have been greatly shortened as suggested; as a consequence, several panels have been removed or moved to Supplementary Material, and former Figures 1 and 2 have been fused to one.

Regarding the possible involvement of iron itself in the rhythmic translation of IRE-mRNAs, we address this point in detail just below, in the answer to Reviewer 2.

# Reviewer #1 (Significance (Required)):

Previous studies have reported a role for iron in altering gene expression and circadian rhythms in mice. The current manuscript extends these studies to show that several IRE-containing mRNAs in liver and IRP2 are subjected to rhythmic regulation. These findings will be interest to researchers in circadian rhythm and iron metabolism fields.

**Author response:** In summary, we are very grateful to the Reviewer for the very positive evaluation.

# Reviewer #2 (Evidence, reproducibility and clarity (Required)):

In the manuscript entitled "Diurnal control of iron responsive element (IRE)-containing mRNAS through iron regular proteins IRP1 and IRP2 is mediated by feeding rhythms", Nadimpalli et al. uncover and mechanistically dissect how the circadian clock and feeding regulates the expression of proteins involved in iron homeostasis in mice. The authors first utilized RNAseq and ribose data and found that a subset of mRNAs containing IREs display rhythmic translation in the liver and/or kidney. The authors then utilized previously published or newly generated datasets to study the origin of these oscillations. After a careful and thoughtful examination, they determine that the oscillations of those mRNAs in the liver are mainly driven by feeding-associated signals, although they are influenced by other factors.

This is a very careful and detailed study that addresses an important issue. The study is really rigorous and the logic is very well explained. So overall this study is very solid and the main conclusion of the study (that the oscillations of those mRNAs are driven by feeding) is solidly established. However, the study falls short on addressing the mechanism of this regulation and if it is different of other feeding regulated mRNA oscillations. This diminishes the significance of the study unless additional mechanistic details are provided. Also, the manuscript first sections (which mainly describe negative results) seem too long and descriptive. Still this is an important and solid study.

**Author response:** We thank the Reviewer for their generally positive evaluation of our manuscript.

Regarding the issue of the molecular cue, and in particular a possible involvement of iron itself in the rhythmic translation of IRE-mRNAs, in our previous version of the manuscript we had established the link to feeding, yet we had indeed not identified the precise molecular cue that would underlie the rhythmic regulation. We did discuss the molecular candidates quite extensively in the Discussion section of the manuscript, in particular free, bioactive Fe(II) (forming the so-called “labile iron pool”, or LIP); oxygen; reactive oxygen species. We agree that it remains an important question whether the observed diurnal control could be mediated directly by changes in intracellular iron availability.

Briefly, in the Revision Plan submitted to Genome Biology concomitant with manuscript transfer from Review Commons, we had proposed to use liver pieces from the two relevant timepoints of our study (i.e., ZT5 and ZT12) and apply a method that allows the separate quantification of Fe(II) and Fe(III) (Abcam iron assay ab83366), thus giving insights into whether rhythmicity would correlate with iron levels. We have now carried out these experiments and included the data as Figure 6E. We saw a minute tendency to higher Fe(II) at ZT5 vs. ZT12, but it did not reach statistical significance. Such results suggest diurnal regulation of IRP targets is not caused by fluctuations in Fe(II) levels. However, it remains difficult to draw a very strong conclusion from this experiment for methodological reasons. Thus, the Fe(II) that is measured in this assay includes the LIP (that we are interested in) but also Fe(II) present in various Fe-S-cluster-containing proteins. To the best of our knowledge, there is no

straightforward technology that would allow separating signals from the two pools. Therefore, we can still neither exclude nor validate the model that fluctuations in bioactive Fe(II) regulate IRP1 and IRP2 in a diurnal fashion. We present this data in the revised version of our manuscript with all the attached caveats, as we think it is a relevant analysis for the reader and may motivate researchers in the future to revisit diurnal Fe(II) levels when adequate technology is available.

# Reviewer #2 (Significance (Required)):

The main issue this reviewer has with the manuscript is the significance. Without additional mechanistic insights suggesting that there is something particular different about the regulation of these mRNAs the manuscript is not of extremely high significance.

**Author response:** We present the iron measurements as a first step towards possible mechanistic insights and are very explicit about its limits. We hope that in the future suitable approaches will be developed to measure in vivo in animal tissues the bioactive iron pool that is relevant for the regulatory mechanisms.

# Reviewer #3 (Evidence, reproducibility and clarity (Required)):

The present manuscript highlights the previously neglected component of diurnal rhythms into the study of iron regulation in the liver, a key organ in the systemic regulation of the metal. A major, well substantiated finding is that IRP1 takes over from IRP2 as a highly relevant regulator during a time of maximal use of the combined system. The presence of a "dietary signal" that sustains the cycling of cellular IRE binding activity in the liver, although undisputable, is perhaps a lesser claim until these or other investigators can confirm or refute the possibility that iron itself (i.e., the best-established factor affecting cellular IRE-binding) is such a signal. If iron does the job, then the interest lies in showing diurnal rhythmicity of its availability in the circulatory system, presumably linked to dietary iron absorption. There is plenty of clinical evidence of this in humans, but the present study along with the cited preprint by Bennett et al. appear to be the first demonstrations of this diurnal variation in mice.

The manuscript has other strengths not immediately evident from the above claims made in the abstract. It contains an elegant balance of reanalyzing and reassessing "big data" produced by the same laboratory in the past in light of new experimental findings that have appeared in the meantime in the literature together with important new additions of such data from combined RNAseq and ribo- seq collections using IRP1 and IRP2 knockout animals and, importantly, by making use of published material from other studies that have provided relevant comparators from other knockouts that studied aspects of liver circadian biology. The approach, besides providing robust testing of the ideas presented, opens the field to questions that remain unanswered but seem highly relevant (I come to these below). The authors write in a very open manner not only about the new findings but also about aspects we do not understand, and their systems biology approach is likely to generate new hypotheses to address incognita.

Let me therefore be clear upfront that the response that follows is written on the premise that I evaluate the work presented as ready for publication: The figures have been constructed with care summarizing a lot of careful investigations and the main conclusions derive seamlessly from the experimental data. Rather than taken as potential criticism to the authors, I would ask that the counterviews or limitations that may arise from my response to the paper are better taken as a celebration of the work - and views - presented. Such a discussion is only possible due to the open

style of this communication mentioned above and is meant to provoke a dialogue or even drive further questioning of the datasets and the design of future experimental approaches. If the authors find any of these comments useful for their revision, they are welcome to take them onboard, but everything that follows should be read under the term "optional".

**Author response:** We are grateful to Dr. Missirlis for his support and great appreciation of our work.

One nuance in the field of circadian biology is that a rhythm is deemed to be genuinely "circadian" when it continues in the absence of zeitgebers. In this sense, although all experiments are valuable, the "collapse" of the rhythm in the paradigms where dietary rhythms have been disrupted makes the phenomenology a candidate "epiphenomenon" rather than being closer related to the biological clock(s). Likewise, in the manuscript we never learn how the liver IRE-binding activity behaves in constant darkness.

**Author response:** We thank the referee for raising this important question. It is true that constant (darkness) conditions are used to call a phenomenon circadian. We would nevertheless argue that for a rhythmic feature that is specifically found in liver, the constant darkness definition to distinguish circadian from non-circadian is not fully valid because even in constant darkness, the liver clocks are not in a free-running state but continue to be entrained by the SCN clock (it is only the latter that is free- running under these conditions).

In our manuscript, we actually suggest that the observed rhythms are not a core output of the circadian machinery (Fig. 6 of our manuscript), but indirectly engendered through feeding rhythms, which are coupled to sleep-wake cycles and thus connect in an indirect way to the central circadian clock activity in the SCN.

In wild-type mice we would therefore expect that irrespective of constant darkness or light-dark entrainment (and assuming *ad libitum* feeding), the hepatic rhythms of the relevant IRE-containing transcripts would persist in a similar fashion.

Where the authors mention in a parenthesis "moreover, there are documented links between iron and the circadian timekeeping mechanism itself", I invite them to take a closer look to the paper Konstantinos Mandilaras and I coauthored in 2012 "Genes for iron metabolism influence circadian rhythms in Drosophila melanogaster". In that work, we showed that RNA interference of genes that are required for iron sulfur cluster formation (including on IRP1) in the central clock neurons of the fly result in loss of the circadian rhythm when flies were kept at constant darkness (not so when they were kept under light:dark oscillation). So this point should probably remain open.

Given the phenotypes collected from RNAi in different cell types, we became sensitive to the notion that different cell types may work with different sets of what we might call collectively "iron metabolism genes". Thus, while reading the present differences between the relative contributions of IRP1 and IRP2 in mouse liver at different times during the day (and night), I kept wondering if both function in the hepatocytes or whether the macrophages or other cell types in the liver may have their own particular contributions.

**Author response:** We would like to thank the Reviewer for pointing out this interesting connection that indeed fits well into the framework of our manuscript. We have cited the work by Mandilaras et al. in the context of Figure 3, where we assess *in vivo* and in tissue explants whether IRP-deficiency affects the clock itself.

To follow Reviewer 3’s idea, we have gone a little further in our analyses of around-the-clock expression data to see if any of the components of the Fe-S assembly machinery is rhythmic itself, which could

have the potential to add novel information. Briefly, we have used for this purpose our around-the-clock RNA-seq and ribo-seq data from Janich et al., 2015 (PMID 26486724). In summary, we find that the expression at RNA and/or footprint level is non-rhythmic for the vast majority of genes involved in Fe-S biogenesis, assembly or transport, with the exception of low-amplitude rhythms for *Glrx5* and *Iba57* (Rebuttal Figure 2).

**Rebuttal Figure 2:** Only two genes involved in Fe-S cluster assembly show moderately rhythmic RNA and/or footprint expression, namely *Glrx5* and *Iba57*, according to our datasets from Janich *et al.* (2015), see PMID 26486724. GLRX5 is important for transfer of 2Fe-2S clusters and the maturation of both 2Fe-2S and 4Fe-4S proteins, whereas *Iba57* acts in 4Fe-4S protein maturation (reviewed in PMID 35883565). Interestingly, GLRX5 deficiency stimulates IRP1 RNA-binding activity and increases IRP2 levels (PMID 20364084). The observed rhythm in GLRX5 biosynthesis may thus connect to the observed IRE-mRNA rhythmicity described in our manuscript; however, any mechanistic interpretation would be highly speculative at this point and therefore not suitable for inclusion in our manuscript.

By contrast, all of the following other genes are non-rhythmic throughout (list of Fe-S-relevant genes from a recent review by Shi et al., PMID34660592): Cytoplasmic/nuclear, all non-rhythmic: *Cfd1=Nubp2, Nbp35=Nubp1, Ciapin1, Ndor1, Iop1=Ciao3=Narfl, Ciao1, Ciao2b=Fam96b, Mms19, Ciao2a=Fam96a;* mitochondrial, all non-rhythmic: *Iscu, Nfs1, Isd11=Lyrm4, Acpm=Ndufab1, Fdx1, Fdx2=Fdx1l, Fxn, Hspa9 Hsc20=Hscb, Abcb7, Alr=Gfer, Isca1, Isca2, Nfu1.*

As this analysis yielded mainly “negative results”, and as we are at present unable to propose a solid possible mechanistic link between the *Glrx5* and/or *Iba57* rhythms and our observations of IRP-target genes rhythmicity, we did not include it in our manuscript, but are only putting it for the record into this rebuttal.

Another issue raised by the authors early on relates to the differential effects of IRP1/2 "activation" on different IRE-containing transcripts. This is a fascinating problem, not answered by the six transcripts shown in figure 1G, but I consider that the present paper offers a great service to the field in figure 5I, where an even more comprehensive grouping of IRE-containing transcripts is provided in terms of their "regulation" by IRPs. Future research should attempt to discover features that correlate within the sets of transcripts, as grouped in here.

Thus, another point made repeatedly by the authors that despite four decades of work on the IRPs we still have open questions about how they "regulate" is well taken. In the same tone, their results in relation to IRP2 degradation show that the story of how the presence of iron leads to the degradation of IRP2 has not been fully elucidated, either.

**Author response:** We are most grateful to the Reviewer for having proposed so many insightful ideas and thoughts regarding future work that should be done in this field.

**Referees cross-commenting**

Reading the comment from Reviewer #2 over the lack of a mechanism to explain why only four transcripts with IREs amongst a larger pool are subject to circadian regulation by IRPs somehow reduces the significance of the study, one has to agree that a discovery - likely another component in the system - is wanting. I remain of the view that the present work exposes this "weakness" of the entire field in a global as opposed to a partial manner and in doing so, makes a significant contribution, especially by further sub-classifying the IRE-containing transcripts according to their responsiveness in the diurnal occupancy of their IREs.

I would like to reinforce the comment of reviewer 1 with respect to the antibodies used in this study that should be made available to the community, given the specificity described. My congratulations to the authors.

**Author response:** A description of the antibodies and the antigen used for immunization can now be found in the Methods section.

# Reviewer #3 (Significance (Required)):

A lesson, perhaps, for the field is that sometimes more than one mechanism may be at play in different cellular or physiological contexts, while vigorous testing requires time and resources and we should value examples of such care and openness, an example of which is offered, in my view, by the present study.

Fanis Missirlis

**Author response:** We thank the Reviewer for his very constructive approach and for the generous sharing of his knowledge of the literature and open questions in this field.

**Second round of review**

**Reviewer 1**

This thorough study will be of interest to researchers in circadian rhythm and iron fields.

**Reviewer 3**

The manuscript was previously reviewed and the authors considered carefully all comments. Their paper represents a solid contribution to the field of circadian and/or metabolic (feeding-related) rhythms of iron-related post-transcriptional regulation through iron regulatory proteins in the mouse liver.
